# A Machine Learning-Based Identification of Genes Affecting the Pharmacokinetics of Tacrolimus Using the DMET^TM^ Plus Platform

**DOI:** 10.3390/ijms21072517

**Published:** 2020-04-04

**Authors:** Jeong-An Gim, Yonghan Kwon, Hyun A Lee, Kyeong-Ryoon Lee, Soohyun Kim, Yoonjung Choi, Yu Kyong Kim, Howard Lee

**Affiliations:** 1 Department of Transdisciplinary Studies, Graduate School of Convergence Science and Technology, Seoul National University, Seoul 16229, Korea; vitastar@korea.ac.kr (J.-A.G.); yonghankwon0@gmail.com (Y.K.); lha2000@snu.ac.kr (H.A.L.); kyeongrlee@kribb.re.kr (K.-R.L.); skim196@snu.ac.kr (S.K.); 2 Medical Science Research Center, College of Medicine, Korea University, Seoul 02841, Korea; 3 Department of Biostatistics and Computing, Yonsei University Graduate School, Seoul 03722, Korea; 4 Department of Clinical Pharmacology and Therapeutics, Seoul National University College of Medicine and Hospital, Seoul 03080, Korea; 5 Laboratory Animal Resource Center, Korea Research Institute of Bioscience and Biotechnology, Ochang, Chungbuk 28116, Korea; 6 GC Pharma, Yongin 16924, Korea; choistella321@gmail.com; 7 Daewoong Pharmaceutical Co., Ltd., Seoul 06170, Korea; anace89@gmail.com; 8 Department of Molecular Medicine and Biopharmaceutical Sciences, Graduate School of Convergence Science and Technology, Seoul National University, Seoul 03080, Korea

**Keywords:** decision tree, random forest, machine learning, tacrolimus, genotype

## Abstract

Tacrolimus is an immunosuppressive drug with a narrow therapeutic index and larger interindividual variability. We identified genetic variants to predict tacrolimus exposure in healthy Korean males using machine learning algorithms such as decision tree, random forest, and least absolute shrinkage and selection operator (LASSO) regression. *rs776746* (CYP3A5) and *rs1137115* (CYP2A6) are single nucleotide polymorphisms (SNPs) that can affect exposure to tacrolimus. A decision tree, when coupled with random forest analysis, is an efficient tool for predicting the exposure to tacrolimus based on genotype. These tools are helpful to determine an individualized dose of tacrolimus.

## 1. Introduction

Tacrolimus, a widely-used immunosuppressive agent that prevents acute rejection after organ transplantation. Since the therapeutic index of tacrolimus is narrow and its pharmacokinetic profile varies widely among patients, the U.S. Food and Drug Administration (FDA) recommends individual dose titration and therapeutic drug monitoring for tacrolimus [1]. Therefore, identifying factors including genetic variants that affect the pharmacokinetic variability of tacrolimus may be beneficial for its optimal use.

Several single nucleotide polymorphisms (SNPs) have been previously associated with tacrolimus metabolism [2,3,4,5,6,7]. For example, *rs776746*, also known as 6986A>G, encodes the nonfunctional CYP3A5*3 allele of the *CYP3A5* gene. CYP3A5*3 induces alternative splicing, then protein truncation, resulting in decreased enzymatic activity of CYP3A5. In contrast, transplant patients with fully functional homozygous CYP3A5*1 alleles require a larger dose of tacrolimus to maintain its immunosuppressive effect than those having one or two CYP3A5*3 alleles [6,7,8,9].

The decision tree, a machine learning-based classification tool, is used to group input variables [10,11,12]. A decision tree provides an acyclic (i.e., tree-like classification chart), which consists of branches (or vertices) and nodes (or leaves). A branch denotes a test or set of tests to be performed on a specific property such as genotype while a node indicates a category or class such as phenotype. Decision trees adequately classify patients by their genotypes to diagnose a disease and to predict its prognosis [12,13,14]. Furthermore, random forests integrate or ensemble multiple randomly chosen decision trees, thereby forests, with each decision tree providing an independent classification prediction. The random forest predicts phenotypes from genotypes with a better accuracy than other methods [12,15,16].

We previously reported the results of two clinical studies with tacrolimus [17,18], in one of which we also performed a pharmacogenomic analysis to identify genotypes that altered the pharmacokinetics of tacrolimus [17]. In that study, the least absolute shrinkage and selection operator (LASSO) regression method was used.

In the present study, we expand our pharmacogenomic database by pooling genotype information obtained from another clinical study with tacrolimus [18] to further identify and evaluate genetic variants that could influence the pharmacokinetics of tacrolimus in healthy adult males. To this end, three machine learning algorithms are used, namely decision tree, random forest, and LASSO, and their results are compared. Additionally, in silico binding analyses are performed for the SNPs in the three prime untranslated regions (3′UTRs).

## 2. Results

### 2.1. Subjects

A total of 81 males (42 and 39 in studies A and B, respectively) were enrolled and completed the entire study as planned. The mean ± standard deviation of age, height, body weight, and body mass index in subjects were 27.1 ± 6.1 years, 173.7 ± 5.5 cm, 68.2 ± 6.9 kg, and 22.6 ± 2.1 kg/m^2^, respectively.

### 2.2. Genetic Associations with Tacrolimus Pharmacokinetics by Decision Tree, Random Forest, and LASSO Analyses

**Figure 1:**
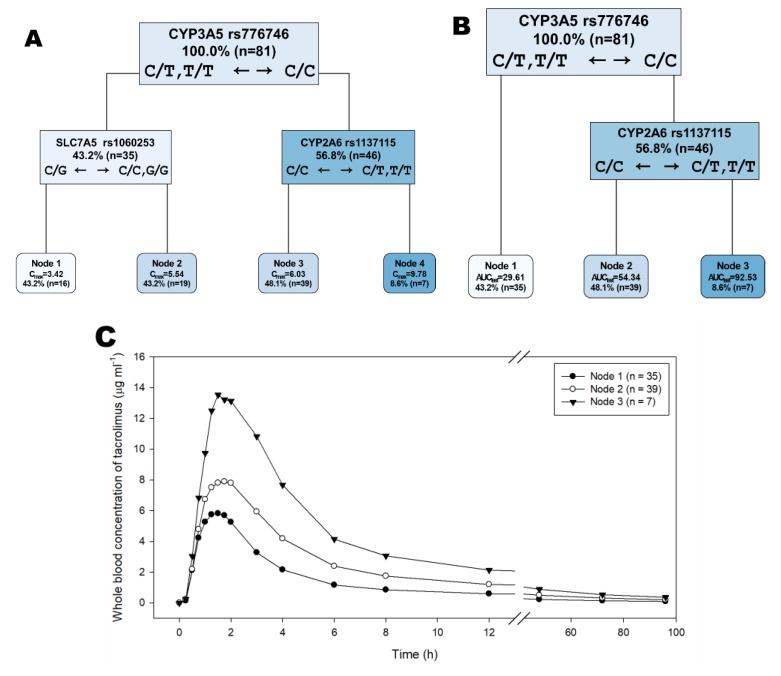
Simplified (depth: 3) decision tree for the maximum plasma concentration (C_max_, μg mL^−1^, **A**) and the area under the concentration curve from time zero to the last quantifiable time point (AUC_last_, h μg mL^−1^, **B**) of tacrolimus. The rectangles denote the branches, which contain the gene name, the single nucleotide polymorphism (SNP) accession number, proportion (%), and frequency of subjects, and the classifying alleles. The rounded rectangles represent the final nodes, in which the mean values of C_max_ and AUC_last_, the percentage, and number of subjects are shown. (**C**) Mean concentration time profiles of tacrolimus by node for AUC_last_ as identified in (**B**). Subjects in node 3 had the highest values of C_max_ and AUC_last_.

The decision trees identified *rs776746* (CYP3A5) as the most important classifying genetic variant for both C_max_ (maximum plasma concentration) and AUC_last_ (area under the concentration curve from time zero to the last quantifiable time point) of tacrolimus, followed by *rs1137115* (CYP2A6) and *rs1060253* (SLC7A5, C_max_ only) (Figure 1A,B; Table 1) when the depth of the decision tree was set to three based on the lowest cross validated (X-val) relative error in C_max_ and the second lowest X-val relative error in AUC_last_ (Figure S1). As a result, the geometric mean C_max_ and AUC_last_ of tacrolimus were 2.36 (95% confidence interval or CI: 1.75–3.18) and 3.40 (95% CI: 2.48–4.66) times greater, respectively, in those carrying the homozygous variant allele for *rs776746* and the reference or heterozygous variant allele for *rs1137115* (node 3 in Figure 1B) than in those carrying the reference or heterozygous variant allele for *rs776746* (node 1 in Figure 1B,C). *rs776746* was also identified as the genetic variant in the random forest analysis that classified both C_max_ and AUC_last_ of tacrolimus with the highest importance (Table 2). Similar to the decision tree analysis, *rs1060253* (SLC7A5) was one of the four high-importance genetic variants for C_max_ in the random forest, whereas *rs1137115* (CYP2A6) was identified as a genetic variant with a high importance for AUC_last_ of tacrolimus (Table 2). Lastly, *rs776746* was the only significant SNP associated with both C_max_ and AUC_last_ of tacrolimus in the LASSO models with a coefficient >0 (Table 3). However, neither *rs1137115* (CYP2A6) nor *rs1060253* (SLC7A5) was retained in the final LASSO models with their coefficients >0. *rs1208* (NAT2) remained in the final LASSO model for C_max_, but the variant allele frequency for *rs1208* was disproportionately higher in our subjects than in the 1000 Genome Projects (Table 3).

### 2.3. In silico Analysis of the SNPs in the 3′UTR

**Figure 2:**
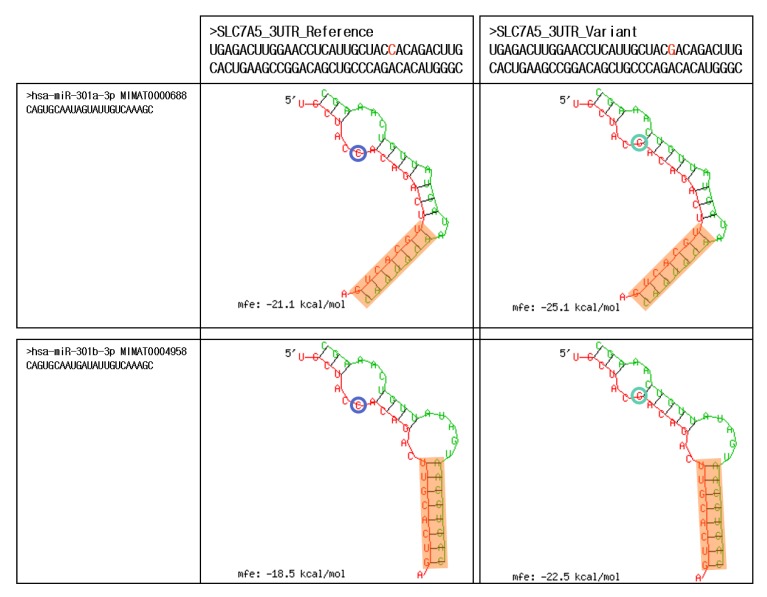
Duplexes identified by in silico analysis between a microRNA (miR) and *rs1060253* of the *SLC7A5* (left: reference allele; right: variant allele) for hsa-miR-301a-3p (top) and miR-301b-3p (bottom). The shades denote the seed region of miR-301a-3p and -301b-3p. The circles represent the reference and variant nucleotides of *rs1060253*.

Of the eight SNPs identified by decision tree, random forest analysis, or LASSO regression (Table 1, Table 2 and Table 3), one SNP (i.e., *rs1060253* of the *SLC7A5*) was located in the 3′UTR (Table 1). Eight miRNAs (miR-130a-3p, -130b-3p, -148a-3p, -148b-3p, -152-3p, -301a-3p, -301b-3p, and -454-3p) had complementary sites for *rs1060253* (Figure 2 and Figure S2). Among these, two miRNAs (miR-301a-3p and -301b-3p) showed different hybrid structures between the reference and variant alleles of *rs1060253* (Figure 2). In contrast, the other six miRNAs had similar hybrid structures between the reference and variant alleles of *rs1060253* (Figure S2).

## 3. Discussion

We demonstrate that *rs776746* (CYP3A5) is consistently the best predictor of exposure to tacrolimus no matter what machine learning algorithms are applied. The evidence is that *rs776746* was repeatedly selected as the most influential genotype in all of the analysis methods employed in this study such as decision tree (Figure 1A,B; Table 1), random forest (Table 2), and LASSO regression (Table 3). Consequently, those carrying the homozygous variant alleles of *rs776746* (i.e., C/C) had a two- to three-times higher AUC_last_ of tacrolimus than those with wild type (T/T) or heterozygous variant alleles (C/T) (Figure 1C). *rs776746* or CYP3A5*3 is located in the terminal sequence of the *CYP3A5′*s intron 3 (Table 1) and induces a premature termination codon. Therefore, subjects carrying *rs776746* have an increased systemic exposure to tacrolimus caused by the reduced metabolism of tacrolimus by *CYP3A5* as shown in the present studies [19,20,21,22,23,24].

Other SNPs had a relatively smaller and inconsistent effect on the systemic exposure to tacrolimus. Of these, however, *rs1137115* in the *CYP2A6* gene is noteworthy although it was not identified in our previous study [17] or by the LASSO regression in the present study (Table 3). Namely, when the reference or heterozygous allele for *rs1137115* was combined with the homozygous variant allele for *rs776746*, the systemic exposure to tacrolimus was much higher than with the homozygous variant allele for *rs1137115* (Figure 1A,B). Additionally, the C/C genotype of *rs1137115* was identified as one the four high-importance genetic variants to classify AUC_last_ (Table 2). The *CYP2A6* gene plays an important role in nicotine metabolism, and *rs1137115* is a regulator of alternative splicing [25,26]. *rs1137115* is associated with lower mRNA expression and reduced nicotine metabolism [25]. However, the observed effect of *rs1137115* on the systemic exposure to tacrolimus is mechanistically hard to explain and is most likely to be a chance finding because the effect is not consistent by the *rs776746* allele (Table S1). *rs3814055* (*NR1I2*) was significantly associated with both C_max_ and AUC_last_ in the false discovery rate (FDR)-adjusted multiple testing analysis and LASSO models in our previous study [13]. However, *rs3814055* was identified as a significant genetic variant for AUC_last_ only by the random forest analysis in the present study (Table 2). Therefore, the role of *rs3814055* should be further confirmed and validated in future studies, preferably in patients. Likewise, the role of *rs1208* (NAT2) is rather inconclusive because most of our subjects carried the variant allele for this SNP.

miRNAs are a transcriptional inhibitor, which recognizes the specific seed regions in the 3′UTR sequences [27], thereby suppressing gene expression [28]. *rs1060253* (*SLC7A5*) is located in the 3′UTR [29,30]. Therefore, genotypic variations in *rs1060253* could change the target sites for hsa-miR-301a-3p and -301b-3p in *SLC7A5* 3′UTR (Figure 2), which could contribute to the altered metabolism of tacrolimus. Genetic variant frequencies of *rs1060253* (SLC7A5) were different between the populations included in the 1000 Genomes Project, and our frequency pattern was like that in Japanese patients as well. The ethnic differences in SLC7A5 are affected by natural selection, migration, and genetic drift, and verifying these differences will help us better understand the ethnic variations in drug susceptibility and phenotypes.

Several previous studies adopted various machine learning algorithms, such as support vector machine [12,31], neural network [32], decision tree [12], and random forest [12], to assess the effect of genetic variations on tacrolimus pharmacokinetics. In those studies, subjects with renal transplantation [12,32] or liver transplant recipients [31] were investigated. The present study is different from those previous studies. First, our subjects are healthy, not transplanted patients [6,7]. This could be beneficial in that the relationships between genetic variations and tacrolimus pharmacokinetics were not confounded by many disease-related variables, which could not be easily adjusted for in many cases as previously shown [13]. Furthermore, we demonstrate that *rs776746* (CYP3A5) is consistently the best predictor of exposure to tacrolimus no matter what machine learning algorithms are used (Table 1, Table 2 and Table 3). This finding is important in that *rs776746* seems to be the most important genetic variation to characterize the exposure to tacrolimus in heterogenous groups of transplant recipients in large, diverse populations.

The present study has several limitations. First, the sample size was relatively small, and all the subjects were healthy males. Therefore, any genetic variants for tacrolimus exposure found only in females or transplant patients could not be detected. Some CYP gene families, renal or hepatic transporters have different expression patterns between males and females [33]. Furthermore, the pharmacokinetics profiles of tacrolimus were slightly different between healthy individuals and transplant patients [34]. Second, although the subjects were collected as a homogenous population, some variations in age, body weight, and body mass index were not evitable, which was not considered in our analyses. Lastly, all the variants detected in this study were limited to those the DMET^TM^ (Drug metabolism enzymes and transporters) provides. Further larger pharmacogenomic studies in transplant patients with tacrolimus are warranted to validate our findings.

In conclusion, *rs776746* (CYP3A5) and *rs1137115* (CYP2A6) were identified as SNPs that could affect the exposure to tacrolimus. A decision tree, when coupled with random forest analysis, is an efficient tool for classifying or predicting the exposure to tacrolimus based on genotype, which is indispensable for its optimal dose selection.

## 4. Materials and Methods

### 4.1. Clinical Studies and Subjects

Study A was a bioequivalence trial of a generic tacrolimus (Tacrobell^®^, Chong Kun Dang Pharmaceutical, Seoul, Korea) and its reference product (Prograf^TM^, Astellas Pharma Korea, Seoul, Korea) [17]. Study B compared the pharmacokinetics of a new tablet formulation of tacrolimus (Tacrobell^®^, Chong Kun Dang Pharmaceutical, Seoul, Korea) with those of the reference capsule formulation (Prograf^TM^, Astellas Pharma Korea, Seoul, Korea) [18]. In each study, healthy male volunteers aged 19–55 and 19–45 years, respectively, received a single oral administration of tacrolimus in different products (study A) or formulations (study B), and blood samples were intensively obtained for pharmacokinetics analysis of tacrolimus. All of the subjects in studies A and B gave written consent for further use of their data, which were also reviewed and approved by the Institutional Review Boards at Seoul National University Hospital (IRB No.: H-1307-087-505, 26 Aug 2013 and H-1412-016-631, 24 Nov 2014, respectively).

### 4.2. Determination of Tacrolimus Concentrations and Pharmacokinetic Analysis

Tacrolimus concentrations in whole blood were determined using a validated LC/MS/MS method [17,18,35]. In the present study, we analyzed only the tacrolimus concentrations of the reference product. The observed concentrations were used to decide the maximum concentration (C_max_) of tacrolimus. The area under the concentration curve from time zero to the latest quantifiable time point (AUC_last_) was calculated using the linear trapezoidal method. All the pharmacokinetics parameters were estimated using a non-compartmental analysis option in the Phoenix WinNonlin (version 6.3; Certara USA Inc., Princeton, NJ, USA).

### 4.3. DNA Extraction and Genotype Analysis

Genomic DNA was extracted from whole blood using QuickGene-mini80 (Fujifilm, Tokyo, Japan). Pre-amplified multiplex PCR samples were put into the DMET^TM^ Plus assay flow system (Affymetrix, Santa Clara, CA, USA), which generated nucleotide signals in the Affymetrix GeneChip^®^ Targeted Genotyping System (Affymetrix, Santa Clara, CA, USA). These nucleotide signals were converted to genotypes using the Affymetrix DMET^TM^ Console software (Affymetrix, Santa Clara, CA, USA) by DNA Link (Seoul, Korea). A total of 1876 out of 1946 genetic markers in the DMET^TM^ Plus microarray were successfully assayed (>95% genotyping calls), and the same variants were excluded, resulting in 567 genotypes for analysis. In addition, we calculated the proportions of reference and variant alleles for identified genotypes in subjects, and compared them with the results from the 1000 Genomes Project [36].

### 4.4. Statistical Analysis and Machine Learning Application

We used three machine learning algorithms: decision tree, random forest, and LASSO. First, the classification and regression trees (CART) algorithm was used to classify subjects based on the 567 genetic variants involved in tacrolimus metabolism and transport. The CART algorithm is helpful for partitioning the data space, then fitting a prediction model within each partition [37]. The partitions were designed as a binary decision tree. The number of splits in the decision trees were predicted by the different complexity parameter and its corresponding cross validated (X-val) relative errors. The X-val relative errors were calculated by 10-fold cross validation [38]. Second, a random forest analysis was performed using 1000 bootstrap samples from the original data set with 43 splitting variables, which was determined as the elbow point in the replicated training processes with 950 predictors of 81 samples. Then, we derived Gini Importance for each classifying genotype. Gini Importance, defined as the total decrease in node impurity averaged over individual decision trees in the random forest, is a measure of each variable’s importance for estimating a target variable [39]. Lastly, a LASSO regression model was fit, and the tuning parameter was decided to minimize the 10-fold cross-validation errors [40]. To obtain an appropriate lambda value of the LASSO regression model, we performed 1000 repetitions, the mode of which was selected.

The decision tree, random forest analyses, and LASSO regression were performed using the R packages rpart, randomForest and glmnet, respectively (version 3.5.1, R Development core team, Vienna, Austria).

## 5. Conclusions

We revealed that *rs776746* (CYP3A5) and *rs1137115* (CYP2A6) can affect exposure to tacrolimus in healthy Korean males using three machine learning algorithms (decision tree, random forest, and LASSO regression). A decision tree and random forest analysis were an efficient tool for predicting the exposure to tacrolimus based on genotype. These methods could be applied to determine an individualized dose of tacrolimus.

## Figures and Tables

**Table 1:** Genetic variants associated with tacrolimus C_max_ and AUC_last_ identified by decision tree.

Gene	SNP	Location	Reference Allele	Variant Allele	Reference Allele Frequency		Variant Allele Frequency	
					1000 Genomes *	Our Data **	1000 Genomes *	Our Data **
*CYP3A5*	*rs776746*	Splice acceptor	T	C	0.379	0.253	0.621	0.747
*CYP2A6*	*rs1137115*	Exon	T	C	0.239	0.136	0.761	0.864
SLC7A5 ^***^	*rs1060253*	3′UTR	G	C	0.698	0.370	0.302	0.630

Abbreviations: C_max_, maximum plasma concentration; AUC_last_, area under the concentration curve from time zero to the last quantifiable time point; SNP, single nucleotide polymorphism. The allele frequency was calculated using the 1000 Genomes Project * data and our data **. SNP data were retrieved from dbSNP. ^***^ C_max_ only.

**Table 2:** Top four genetic variants for tacrolimus C_max_ and AUC_last_ identified in the random forest analysis.

Gene	SNP and Genotype	Location	Reference Allele	Variant Allele	Reference Allele Frequency		Variant Allele Frequency		Importance
					1000 Genomes *	Our Data **	1000 Genomes *	Our data **	
C_max_									
*CYP3A5*	*rs776746*	Splice acceptor	T	C	0.379	0.253	0.621	0.747	0.28524489
*SLCO3A1*	*rs2190748*	Intron	G	A	0.517	0.525	0.483	0.475	0.14800742
*ADC1*	*rs1049793*	Exon	C	G	0.627	0.358	0.373	0.642	0.13512953
*SLC7A5*	*rs1060253*	3′UTR	G	C	0.698	0.370	0.302	0.630	0.11857793
AUC_last_									
*CYP3A5*	*rs776746*	Splice acceptor	T	C	0.379	0.253	0.621	0.747	1.5377314
*SLCO3A1*	*rs2190748*	Intron	G	A	0.517	0.525	0.483	0.475	0.3333521
*CYP2A6*	*rs1137115*	Exon	T	C	0.239	0.136	0.761	0.864	0.1921316
*NR1I2*	*rs3814055*	Exon	C	T	0.678	0.710	0.322	0.290	0.1419874

Abbreviations: C_max_, maximum plasma concentration; AUC_last_, area under the concentration curve from time zero to the last quantifiable time point; NA, not applicable. The allele frequency was calculated using the 1000 Genomes Project * data and our dataset **.

**Table 3:** Genetic variants with a coefficient >0 for tacrolimus C_max_ and AUC_last_ in the least absolute shrinkage and selection operator (LASSO) models.

Gene	SNP	Location	Reference Allele	Variant Allele	Reference Allele Frequency		Variant Allele Frequency		Coefficient
					1000 Genomes *	Our Data **	1000 Genomes *	Our Data **	
C_max_									
*CYP3A5*	*rs776746*	Splice acceptor	T	C	0.379	0.253	0.621	0.747	0.13331
*CBR1*	*rs3787728*	Intron	T	C	0.270	0.519	0.730	0.481	0.07863
*NAT2*	*rs1208*	Exon	G	A, T	0.323	0.025	0.677	0.975	0.07224
AUC_last_									
*CYP3A5*	*rs776746*	Splice acceptor	T	C	0.379	0.253	0.621	0.747	0.36133

Abbreviations: C_max_, maximum plasma concentration; AUC_last_, area under the concentration curve from time zero to the last quantifiable time point. The allele frequency was calculated using the 1000 Genomes Project * data and our dataset **.

## Supplementary Materials

Supplementary Materials can be found at https://www.mdpi.com/1422-0067/21/7/2517/s1.
