# Supplementary material for: A Machine Learning-Based Identification of Genes Affecting the Pharmacokinetics of Tacrolimus Using the DMETTM Plus Platform
Source: Int J Mol Sci. 2020 Apr 4;21(7):2517. doi: 10.3390/ijms21072517 (PMC7178269; doi:10.3390/ijms21072517)
Supplement: Supplementary file 1 [file ijms-21-02517-s001.pdf]

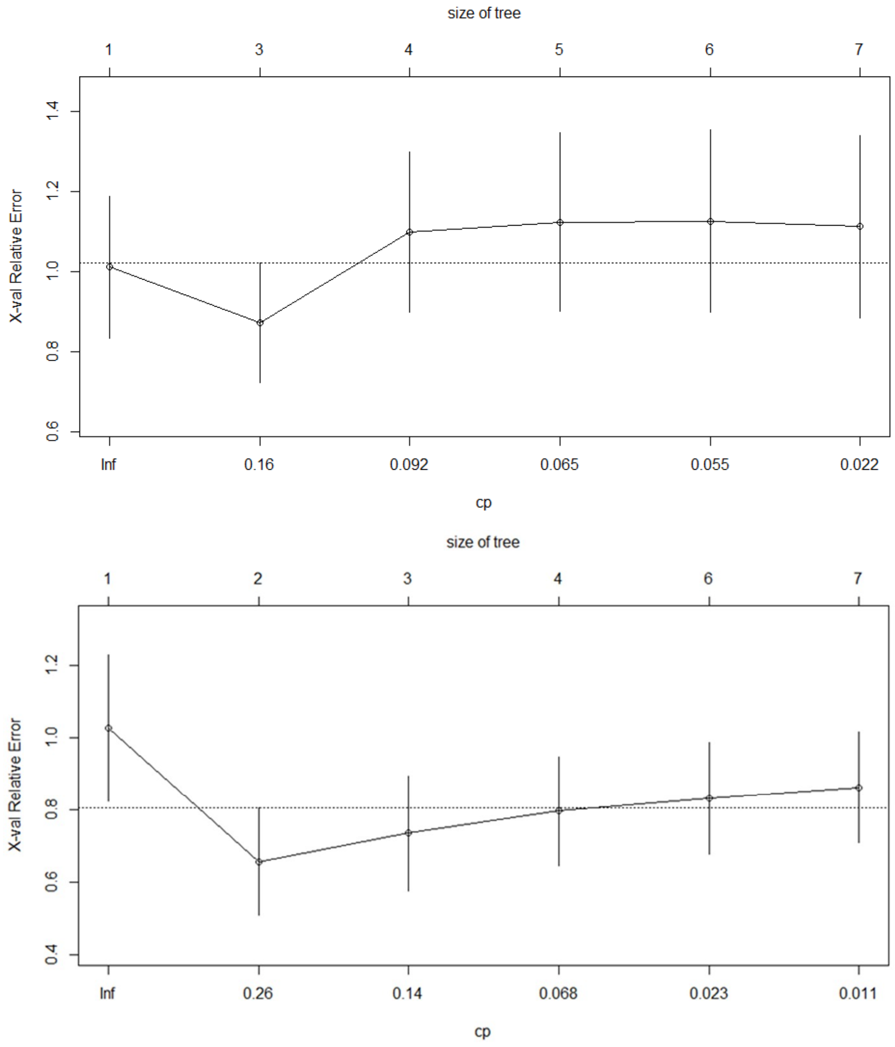

Supplemental Figure S1. Number of splits in the pruned decision trees with different complexity parameters (CPs) and the corresponding cross validated (X-val) relative errors. The horizontal axis is not in scale. The dashed line indicates the minimum relative error.

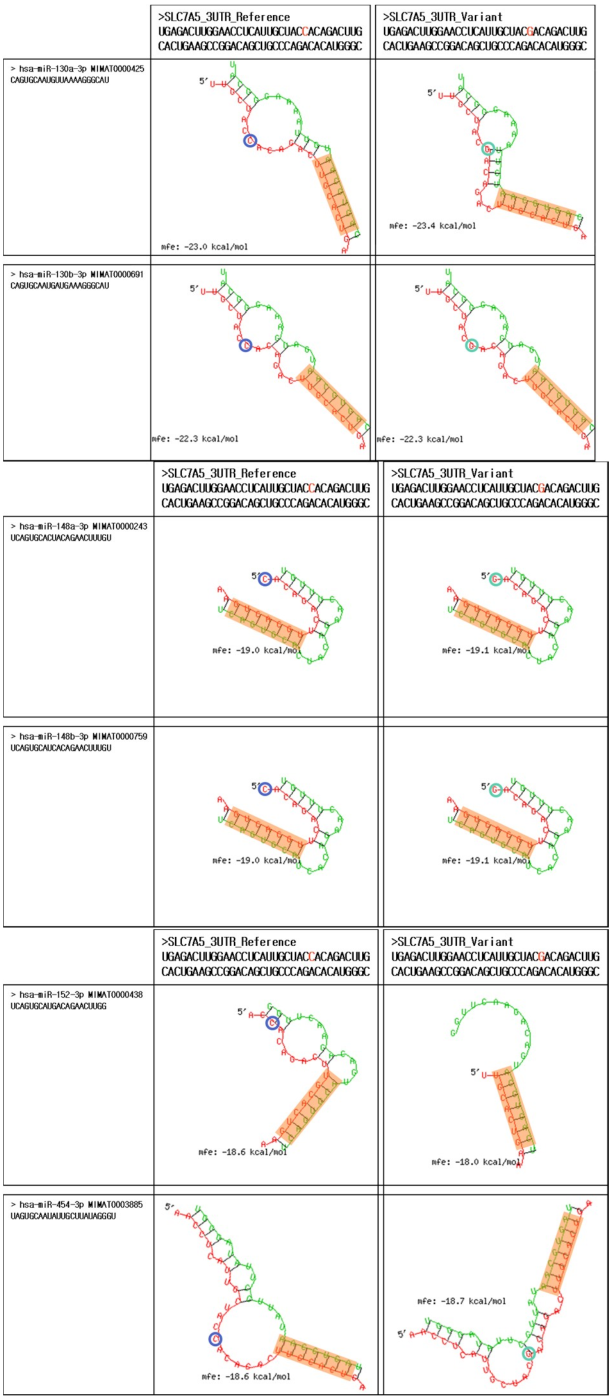

Supplemental Figure S2. Duplexes identified by in silico analysis between microRNAs (miRs) and *rs1060253* of the *SLC7A5* (left: reference allele; right: variant allele) for six miRs (top: hsa-miR-130a-3p, -148a-3p, and -152-3p from left to right; bottom: -130b-3p, 148b-3p, and -454-3p from left to right). The shades denote the seed region of six miRNAs (miR-130a-3p, -130b-3p, -148a-3p, 148b-3p -152-3p, and -454-3p). The circles represent the reference and variant nucleotides of *rs1060253*.

Supplemental Table S1. Tacrolimus C_{max} and AUC_{last} by *rs776746* (*CYP3A5*) and *rs1137115* (*CYP2A6*).

		rs1137115					
		C/C			T/T, C/T		
		Total subject number	C_{max} (μg ml^{-1})	AUC_{last} (h μg ml^{-1})	Total subject number	C_{max} (μg ml^{-1})	AUC_{last} (h μg ml^{-1})
rs776746	C/C	39	6.03±2.27	54.34±21.44	7	9.78±1.92	92.53±20.46
	T/T, C/T	24	4.94±1.41	31.52±8.23	11	3.75±1.06	25.44±7.72

Abbreviations: C_{max}, maximum plasma concentration; AUC_{last}, area under the concentration curve from time zero to the last quantifiable time point.

Data are shown as mean ± standard deviation.
